# Nontargeted metabolomics analysis of follicular fluid in patients with endometriosis provides a new direction for the study of oocyte quality

**DOI:** 10.1002/mco2.302

**Published:** 2023-05-30

**Authors:** Yiqiu Wei, Zhourui Zhang, Yaoyao Zhang, Jianan Li, Xianqin Ruan, Qiongqiong Wan, Tailang Yin, Yujie Zou, Suming Chen, Yan Zhang

**Affiliations:** ^1^ Reproductive Medicine Center Renmin Hospital of Wuhan University Wuhan Hubei China; ^2^ The Institute for Advanced Studies Wuhan University Wuhan Hubei China; ^3^ Department of Obstetrics and Gynecology Key Laboratory of Birth Defects and Related of Women and Children of Ministry of Education, West China Second University Hospital, Sichuan University Chengdu Sichuan China; ^4^ Department of Clinical Laboratory Renmin Hospital of Wuhan University Wuhan Hubei China

**Keywords:** endometriosis, follicular fluid, infertile, LC‒MS, metabolomics, reproductive

## Abstract

Endometriosis is a common, estrogen‐dependent chronic gynecological disease that endangers the reproductive system and systemic metabolism of patients. We aimed to investigate the differences in metabolic profiles in the follicular fluid between infertile patients with endometriosis and controls. A total of 25 infertile patients with endometriosis and 25 infertile controls who were similar in age, BMI, fertilization method and ovulation induction treatment were recruited in this study. Metabolomics analysis of follicular fluid was performed by two methods of high‐performance liquid chromatography tandem mass spectrometry. There were 36 upregulated and 17 downregulated metabolites in the follicular fluid of patients in the endometriosis group. KEGG pathway analysis revealed that these metabolites were enriched in phenylalanine, tyrosine and tryptophan biosynthesis, aminoacyl‐tRNA biosynthesis, phenylalanine metabolism and pyrimidine metabolism pathways. A biomarker panel consisting of 20 metabolites was constructed by random forest, with an accuracy of 0.946 and an AUC of 0.988. This study characterizes differences in follicular fluid metabolites and associated pathway profiles in infertile patients with endometriosis. These findings can provide a better comprehensive understanding of the disease and a new direction for the study of oocyte quality, as well as potential metabolic markers for the prognosis of endometriosis.

## Graphical abstract

This study found differences in follicular fluid metabolites and related pathways in infertile patients with endometriosis. It can provide a better comprehensive understanding and new direction for the study of oocyte quality and potential metabolic markers for the prognosis of endometriosis.

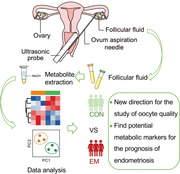

## 1 INTRODUCTION

Endometriosis (EM) is a common, benign, estrogen‐dependent chronic gynecological disease. It is characterized by the presence of endometrial tissue outside its normal position. The main symptoms are pelvic pain and infertility, affecting 8%−10% of women of childbearing age.^1^ It has been reported that the prevalence of endometriosis in women with infertility is as high as 20%−30%.^2^ In the past three decades, assisted reproductive technology (ART) has covered the management of almost all types of infertility. The pregnancy rate of ART in patients with endometriosis, however, is still low,^3^, ^4^ which may be due to the adverse effects of endometriosis on fertilization, embryonic development and implantation.^5^ It has been reported that the pregnancy rate of endometriosis patients receiving donated oocytes is significantly higher than that of those receiving their own oocytes,^6^ indicating that the adverse effect on oocytes may be an important aspect of endometriosis‐related infertility.^7^

Follicles are the basic functional units for the occurrence and development of oocytes. Generally, it can be divided into four stages: primordial follicle, preantral follicle, antral follicle and preovulatory follicle. From the antral follicular phase, follicular cells and follicular membrane vascular secretions fill the antrum, known as follicular fluid (FF). FF constitutes an important microenvironment for oocyte development and maturation which contains important metabolites such as growth factors, cytokines, energy substrates, amino acid steroids, lipids, and cholesterol. These metabolites accumulate in oocytes and are crucial for their growth and development.^8^, ^9^, ^10^

Metabolomics is a useful tool for comprehensively characterizing metabolic changes in different physiological or pathological conditions.^11^ Most of the studies on EM metabolomics are focused on blood,^12^, ^13^ urine,^14^ endometrial fluid,^15^ endometrial tissue,^16^ and other samples.^17^ In recent years, an increasing number of researchers have noticed that FF is the main growth and development microenvironment of oocytes, and its metabolic profile may have an important relationship with the developmental potential of oocytes.^18^, ^19^ Some researchers have used ^1^H‐nuclear magnetic resonance spectroscopy (^1^H‐NMR) to analyze the metabolism of FF samples. It was found that the concentrations of glucose, citrate, creatine and some amino acids in deeply infiltrating endometriosis women were lower than those in the control group, while lactic acid, pyruvate, lipids and ketone bodies were higher.^20^ Castiglione et al.^21^ and Marianna et al.^22^ also found that small molecular metabolites in FF play an important role in oocyte development. Sun et al. found upregulation of some lysophosphatidylcholines (LPCs) in FF using the sequential window acquisition of all theoretical fragment‐ion spectra (SWATH™) method on ultraperformance liquid chromatography (UPLC)–time‐of‐flight (TOF) mass spectrometry (MS),^23^ and Cordeiro et al. also detected that sphingolipids and phosphatidylcholines were more abundant in the EM group by MS‐based lipidomics.^24^ However, the sample size was small and mainly explained the changes in lipids, while differences in other types of metabolites were seldom found. Metabolomics research in this aspect still deserves further research.

In this study, we used a metabolomics method based on liquid chromatography tandem mass spectrometry (LC‒MS) to study the metabolic changes in FF in patients with endometriosis. The specific objectives were as follows: (i) to identify the differential metabolites in the FF of patients with endometriosis and (ii) to study the effect mechanism of FF on oocyte maturation and quality in patients with endometriosis infertility. At the same time, the potential of some differential metabolites to predict in vitro fertilization and embryonic development of oocytes was evaluated. Through this study, we can better understand the disease comprehensively, provide new directions for the study of oocyte quality, and provide potential metabolic markers for epidemiological diagnosis.

## 2 RESULTS

### 2.1 Comparison of baseline clinical characteristics and laboratory data suggests lower quality of embryos in the EM group

First, we analyzed the baseline clinical characteristics and laboratory data of the control group in the EM group. The clinical characteristics, including age, BMI, hormone characteristics, and markers of ovarian reserve (follicle‐stimulating hormone (FSH), anti‐Müllerian hormone (AMH), and antral follicle count (AFC)), are listed in Table 1. There was no significant difference between the EM group and the control group in the baseline clinical characteristics. Among the 25 patients in the EM group, 12 patients (48%) were diagnosed with endometriosis by operation (the EM diagnosis group, EMD), and the rest were suspected to have endometriosis by type B ultrasound (the suspected EM group, SEM). To analyze the difference between the EMD group and the SEM group in FF metabolomics data, univariate analysis and principal component analysis (PCA) were conducted. The volcano plot showed no differential metabolites with false‐discovery rate (FDR) < 0.05 (Figure S1A), and the clusters were completely confused together in the PCA model (Figure S1B). Furthermore, hierarchical cluster analysis was also unable to successfully classify these two subgroups (Figure S1C). These results indicated that there was no difference in the FF metabolic profile between EMD patients and SEM patients.

**TABLE 1:** Baseline clinical characteristics of the EM group and control group.

	EM group (*n* = 25)	Control group (*n* = 25)	*p* Value
Age (years)	31.44 ± 3.58	32.20 ± 3.03	0.42
BMI (kg/m^2^)	21.30 ± 2.68	21.24 ± 1.77	0.93
AMH (ng/mL)	3.10 ± 1.32	3.19 ± 1.67	0.83
Baseline estradiol (pg/mL)	42.94 ± 17.82	40.10 ± 16.23	0.55
Baseline LH (mU/mL)	3.43 ± 1.75	3.34 ± 1.21	0.84
Baseline FSH (IU/L)	7.25 ± 2.25	7.46 ± 2.36	0.75
Progesterone (nmol/L)	0.67 ± 0.54	0.53 ± 0.18	0.15
Fasting blood glucose (mmol/L)	4.93 ± 0.50	5.35 ± 0.96	0.06
AFC (*n*)	13.68 ± 6.52	16.24 ± 5.43	0.46
Years of infertility (years)	2.84 ± 2.07	2.07 ± 1.29	0.15
Number of IVF cycles (*n*)	17	17	–
Number of ICSI cycles (*n*)	8	8	–
Total FSH dose per cycle (IU)	1719.1 ± 495.65	1678 ± 540.33	0.78
Gn days (days)	10.8 ± 2.69	11.32 ± 3.56	0.56

The data are expressed as the number or mean ± SD. There was no significant difference between the endometriosis group and the control group (*p* > 0.05).AFC, antral follicle counting; AMH, anti‐Müllerian hormone; BMI, body mass index; E2, estradiol; FSH, follicle‐stimulating hormone; IVF, in vitro fertilization; ICSI, intracytoplasmic sperm injection; LH, luteinizing hormone; SD, standard deviation.

The laboratory data and the average number of oocytes obtained from the samples are listed in Table 2, where the MII oocyte rate, two pronucleus (2PN) fertilization rate, 2PN cleavage rate and high‐quality embryo rate were counted. As the calculation methods of the in vitro fertilization (IVF) type and the intracytoplasmic sperm injection (ICSI) type were different, they were calculated separately. The IVF type had a higher 2PN fertilization rate, but the difference was not significant. In addition, compared with the EM group, the control group had a significantly higher high‐quality embryo rate (*p* value = 0.036) in the IVF type, which indicates that FF may affect the quality of oocytes. However, for the ICSI type, the difference in the high‐quality embryo rate between the two groups was not significant.

**TABLE 2:** Laboratory data of EM patients and controls.

		Average number of obtained oocytes	MII oocytes rate (%)	2PN fertilization rate (%)	2PN cleavage rate (%)	High‐quality embryo rate (%)
IVF	EM	11.24	72.77 (139/191)	53.40 (102/191)	99.02 (101/102)	49.50 (50/101)
	Control	11.47	68.71 (134/195)	56.41 (110/195)	98.18 (108/110)	63.89 (69/108)
*p* Value		0.91	0.38	0.55	–	0.036*
ICSI	EM	9.13	82.19 (60/73)	66.67 (40/60)	100 (40/40)	37.50 (15/40)
	Control	10.25	74.39 (61/82)	62.30 (38/61)	100 (38/38)	42.11 (16/38)
*p* Value		0.69	0.24	0.62	–	0.68

The data are expressed as *n*% (ratio) or average.

MII oocyte rate = the number of MII oocytes/the number of oocytes obtained.

IVF 2PN fertilization rate = the number of 2PN fertilization/the number of oocytes obtained.

ICSI 2PN fertilization rate = the number of 2PN fertilization/the number of MII oocytes.

2PN cleavage rate = the number of 2PN fertilization/the number of 2PN cleavage.

High‐quality embryo rate = the number of high‐quality embryos/the number of 2PN cleavages. *p < 0.05. The difference is statistically significant.

AFC, antral follicle counting; AMH, anti‐Müllerian hormone; BMI, body mass index; E2, estradiol; FSH, follicle‐stimulating hormone; IVF, in vitro fertilization; ICSI, intracytoplasmic sperm injection; LH, luteinizing hormone; MII, second meiotic division; 2PN, two pronucleus.

*p < 0.05. The difference is statistically significant.

### 2.2 Quality control analysis showed that the metabolomics data were reliable

**FIGURE 1:**
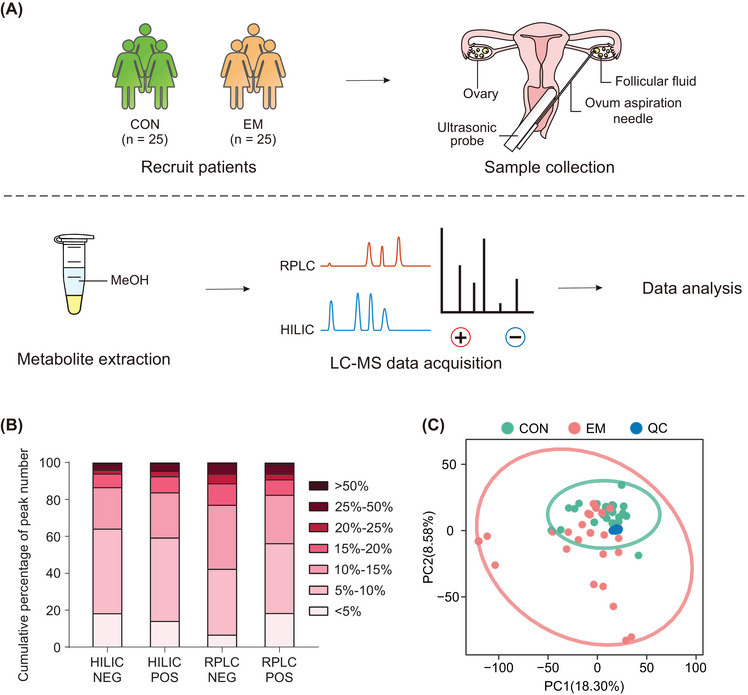
Workflow and data quality analysis. (A) Workflow of this study. The EM group and the control group of infertile patients were recruited to obtain follicular fluid during oocyte collection. The obtained follicular fluid was extracted by MeOH for metabolites, separated by RPLC and HILIC, and detected by positive and negative ion mode mass spectrometry. The detection results were obtained for data analysis. (B) Distribution of RSD values of QC peaks in four modes. (C) Overall PCA of data synthesized in four modes. COM, control group; EM, endometriosis group; HILIC, hydrophilic interaction liquid chromatography; LC‐MS, liquid chromatograph mass spectrometer; MeOH, methanol; QC, quality control; RPLC, reversed‐phase liquid chromatography; +, positive ion mode; –, negative ion mode.

To ensure the reliability of the metabolomics data, we conducted quality control analysis. The workflow of this study is shown in Figure 1A. Before the injection of each mode, the mass‐to‐charge ratio (*m/z*) measurement was externally calibrated by sodium formate. Quality control (QC) samples were injected 10 times before FF samples to balance the instrument. During data acquisition, QC samples were inserted every 10 FF samples. The Pearson's correlation coefficient of QC samples was calculated to evaluate the stability of the serial injections. The results showed that the correlation coefficients were all higher than 0.95 (Figure S2). The peaks of all experimental samples and QC samples were extracted, and PCA was performed after autoscaling. The results showed that QC samples were closely packed together (Figures 1C and S3). The ratio of peaks with the relative standard deviations (RSDs) less than 25% in all QC samples exceeded 90% (Figure 1B). All of these results show that the metabolomics data were reliable.

### 2.3 Metabolic profiling analysis of FF finds metabolic changes in patients with endometriosis

We conducted a series of analyses to investigate the metabolic changes in patients with endometriosis. The RSD value of every feature in QC samples was used to filter the low‐quality data. Features with RSD > 0.25 were excluded.^25^ After filtering the features with missing values greater than 20%, the missing value was filled with 1/5 of the minimum positive value. Normalized peak intensity by total ion chromatogram (TIC) was used for the subsequent analysis. In this study, PCA was constructed to reveal the overall difference in metabolites between the EM group and the control group. Unsupervised pattern recognition PCA, supervised pattern recognition partial least squares discriminant analysis (PLS‐DA) and orthogonal partial least squares discriminant analysis (OPLS‐DA) were used to reduce the dimension of the obtained multidimensional data into a matrix to maximize the separation trend between the two groups. R2Y (fitting effect of the model in the Y matrix) and Q2Y (prediction ability of the model in the Y matrix) were used to evaluate the stability and predictability of the PLS‐DA model and OPLS‐DA model by cross‐validation (permutation test). A Q2Y threshold of 0.5 was used to determine whether the model can be considered to have good (Q2Y ≥ 0.5) or poor (Q2Y < 0.5) prediction ability.

**FIGURE 2:**
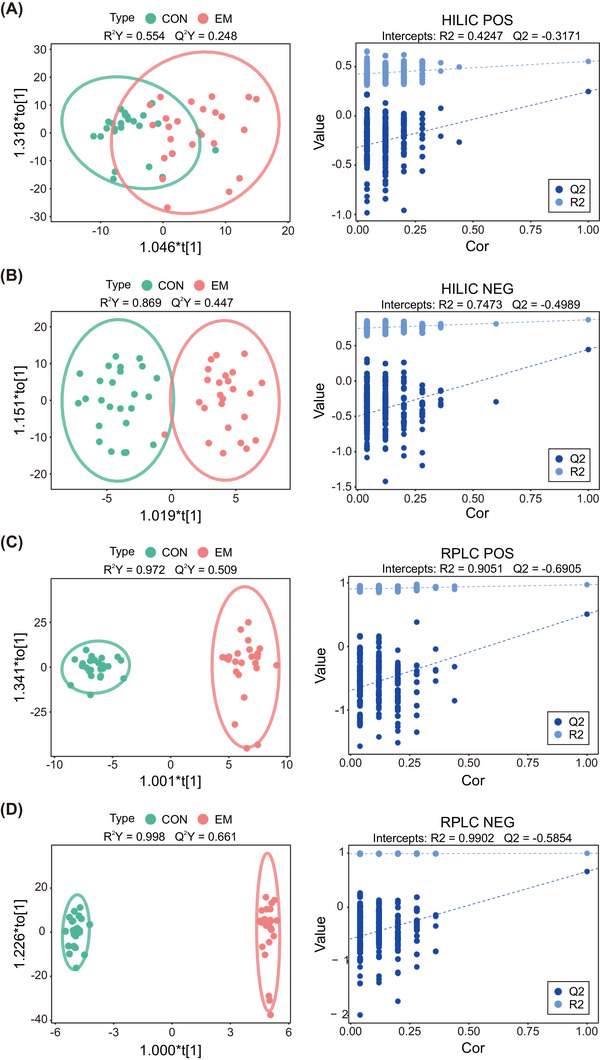
OPLS‐DA scores scatter plot and permutation test describing the trend of separation between the two groups. The EM group and the control group are represented by red and green circles, respectively. Based on the regression model, the OPLS‐DA scores scatter plot shows the separation between the experimental group and the control group. OPLS‐DA scores scatter plot and corresponding model's permutation test of (A) HILIC positive ion mode; (B) HILIC negative ion mode; (C) RPLC positive ion mode; (D) RPLC negative ion mode. Cor, correlation coefficient; OPLS‐DA, orthogonal partial least squares discriminant analysis. R2, fitting effect of the model; Q2, prediction ability of the model.

In the PCA model, green represents the control group, and red represents the EM group. There was no obvious separation between the two groups, which may be due to the complexity and variability of the clinical samples (Figures 1C and S3). The two‐dimensional (2D) score of PLS‐DA shows that there is a separation trend between the control and EM groups (Figure S4). The 2D score of the OPLS‐DA shows that there is obvious separation between controls and EM patients (Figure 2). Through permutation test, it is found that the maximum R2Y is 0.998, and the maximum Q2Y is 0.661, indicating that the model also has a good fitting degree and prediction ability.

**FIGURE 3:**
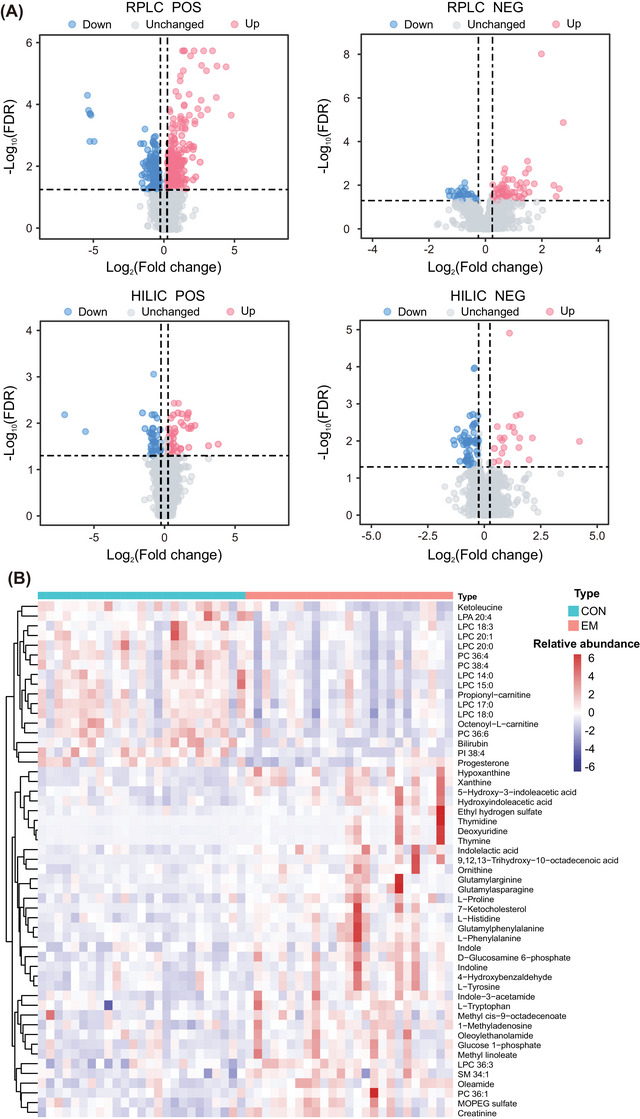
Analysis of differential metabolites. (A) Volcano plot of metabolites in the four modes. The red dots are the metabolites with Log_2_(FC) > 0.25 and FDR < 0.05, and the blue dots are the metabolites with Log_2_(FC) < −0.25 and FDR < 0.05. A total of 207 upregulated features and 114 downregulated features were detected in the positive ion mode of RPLC, and 54 upregulated features and 22 downregulated features were detected in the negative ion mode of RPLC. A total of 53 upregulated features and 21 downregulated features were detected in the positive ion mode of HILIC, and 45 upregulated features and 37 downregulated features were detected in the negative ion mode of HILIC. (B) Heatmap of identified differential metabolites. FC, fold‐change; FDR, false‐discovery rate.

The differentially expressed metabolites between the two groups were selected by fold‐change and *t* test or Mann‒Whitney test. Metabolites with |Log_2_(FC)| > 0.25 and FDR < 0.05 were considered differentially expressed (Figure 3A). As a result, 359 features were upregulated, and 194 features were downregulated in the EM group. After identification, a total of 36 metabolites were found to be upregulated, and 17 metabolites were downregulated in the EM group (Table S1 **;** Figures S5 and **S6**). From the heatmap of these differential metabolites (Figure 3B), we can see clear differences between the two groups, which is mainly manifested by the downregulation of multiple lipids and the upregulation of some metabolites, such as a few amino acids, in the EM group.

### 2.4 Enrichment analysis and correlation analysis revealed that differential metabolites were mainly enriched in four pathways

**FIGURE 4:**
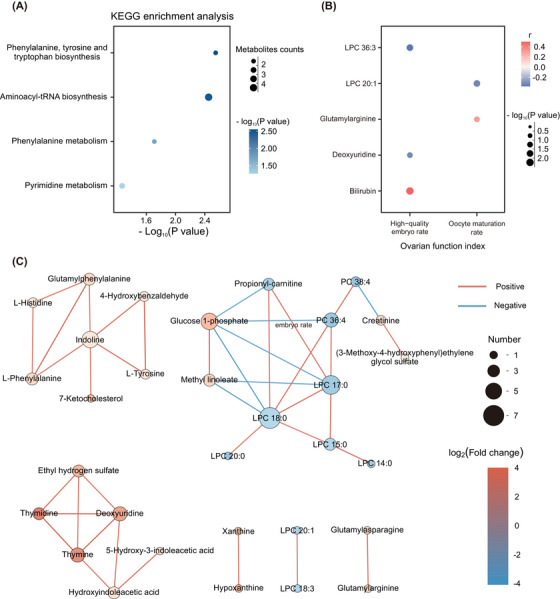
KEGG pathway enrichment analysis and differential metabolite correlation analysis. (A) The enriched KEGG pathways in the EM group. (B) The Spearman's correlation coefficients between differential metabolites and clinical indexes. The size of the point in the map reflects the *p* value, and the color reflects the value of the correlation coefficient *r*. (C) The correlation network diagram of all differential metabolites with *r* > 0.8 (or *r* < −0.8) and *p* value < 0.05. The size of the dot in the figure reflects the number of metabolites associated with the metabolite, and the color reflects the Log_2_(FC) of the metabolite between the EM group and the control group. The color of the line connecting the dots represents a positive correlation (red) or a negative correlation (blue) between the two metabolites. KEGG, Kyoto Encyclopedia of Genes and Genomes.

Enrichment analysis and correlation analysis were conducted to understand the pathways involved in differential metabolites. A total of 26 differential metabolites could be matched in the Kyoto Encyclopedia of Genes and Genomes (KEGG) database. KEGG pathway enrichment analysis showed that these differential metabolites were mainly significantly enriched in phenylalanine, tyrosine and tryptophan biosynthesis (*p* = 0.0028), aminoacyl‐tRNA biosynthesis (*p* = 0.0035), phenylalanine metabolism (*p* = 0.0195), and pyrimidine metabolism (*p* = 0.0538) (Figure 4A; Table S2). The correlation analysis showed that there was an obvious correlation in some metabolites. Figure 4C shows the correlation network of the differential metabolites with a correlation coefficient *r* > 0.8 and *p* value < 0.05. The differential metabolites were mainly divided into four groups (Figure 4C). Group I mainly consisted of some aromatic amino acids and related metabolites, all of which were upregulated in the EM group, indicating that the synthesis of aromatic amino acids may be active. Group II showed that there was a negative correlation between some metabolites and various lipids, indicating that the upregulation of these metabolites may promote the consumption of lipids. Group III mainly included some upregulated purine metabolites in the EM group. Combined with the activation of the pyrimidine metabolism pathway, there may be more active processes, such as cell proliferation and protein synthesis, in the follicular fluid of the EM group. Group IV mainly included 3 groups of metabolites that could be converted to each other and presented a consistent variation trend. A correlation heatmap comprehensively showed the correlation between all differential metabolites, and most metabolites had a negative correlation with lipids (Figure S7).

### 2.5 Correlation patterns were found between differential metabolites and clinical indicators

Different correlation patterns were also found between differential metabolites and clinical indicators. The Spearman's correlation coefficients and their corresponding *p* values are shown in Figure 4B and Table 3. The oocyte maturation rate was positively correlated with glutamylarginine (*r* = 0.3069, *p* = 0.0327) and negatively correlated with LPC 20:1 (*r* = −0.3209, *p* = 0.0164). The high‐quality embryo rate was positively correlated with bilirubin (*r* = 0.4970, *p* = 0.003) and negatively correlated with LPC 36:3 (*r* = −0.3716, *p* = 0.0181) and deoxyuridine (*r* = −0.3143, *p* = 0.049).

**TABLE 3:** Spearman's correlation coefficient between differential metabolites and clinical information in follicular fluid samples.

Metabolites	Ovarian function index	*r*	*p* Value	Type
Glutamylarginine	Oocyte maturation rate	0.3069	0.0327	Positive
LPC 20:1	Oocyte maturation rate	–0.3209	0.0164	Negative
Bilirubin	High‐quality embryo rate	0.4970	0.0030	Positive
Deoxyuridine	High‐quality embryo rate	–0.3143	0.0490	Negative
LPC 36:3	High‐quality embryo rate	–0.3716	0.0181	Negative

### 2.6 Distinguishing endometriosis from controls through machine learning

**FIGURE 5:**
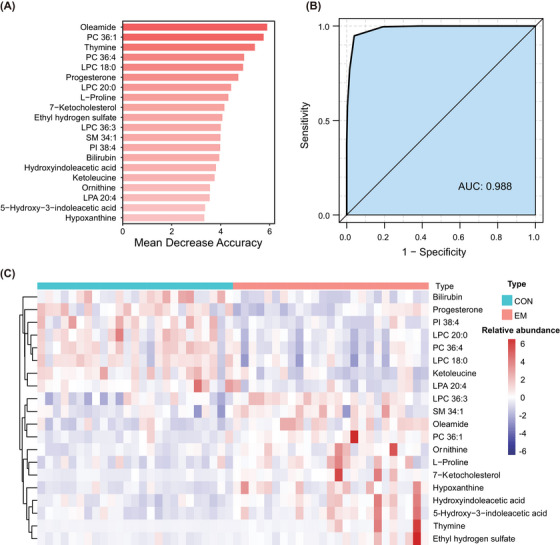
Random forest model and ROC analysis. (A) The rank of importance of 20 differential metabolites for classifying the EM group and the control group. (B) The mean AUC obtained by 10 times fivefold cross‐validation using the top 20 differential metabolites is 0.988. (C) The heatmap including the top 20 differential metabolites shows obvious differences between the two groups. AUC, area under the curve; ROC, receiver operating characteristic.

We used the random forest algorithm to distinguish the EM group from the control group depending on the differential metabolites. We calculated the order of importance of these differential metabolites to classify the EM group and the control group and found that when using the top 20 metabolites, the model had the highest area under the concentration‐time curve (AUC) of 0.988 (Figure 5A and B). The accuracy, sensitivity and specificity were 0.946, 0.948, and 0.944, respectively. The diagnostic ability of the model with the top 20 was better than that with the top 10 and with all differential metabolites (Table S3). The heatmap of these 20 metabolites obviously showed the differences between the two groups (Figure 5C).

## 3 DISCUSSION

It is well known that the quality of oocytes depends in some sense on the follicular microenvironment. Metabolomics under pathophysiological stimulation and environmental or genetic modification has been used for monitoring diseases and developing new treatment strategies. In this study, we observed that a total of 36 metabolites, such as thymidine, thymine, 9,12,13‐trihydroxy‐10‐octadecenoic acid, deoxyuridine, phosphatidyl choline (PC) 36:1, and hypoxanthine, were significantly upregulated in patients with endometriosis. Most of these metabolites were enriched in the pathways of phenylalanine, tyrosine and tryptophan biosynthesis, aminoacyl‐tRNA biosynthesis, phenylalanine metabolism and pyrimidine metabolism. A total of 17 metabolites in the FF of patients with EM were downregulated, most of which belonged to lipids.

The contents of pyrimidine metabolites, such as thymidine, thymine, and deoxyuridine, and purine metabolites, such as xanthine, hypoxanthine and 1‐methyladenosine, were upregulated in the EM group. Purine and pyrimidine are the main components of DNA and RNA, and their upregulation may be related to the increase in adenosine triphosphate (ATP) consumption, DNA replication, transcription and translation. This indicates that there may be more active processes, such as cell proliferation and protein synthesis, in the follicular fluid of the EM group.^26^ Among them, xanthine can be biosynthesized from guanine or by the action of purine nucleoside phosphorylase. Hypoxanthine is a reactive intermediate of adenosine metabolism and nucleic acid formation through nucleotide remediation. In this study, we observed upregulation of xanthine and hypoxanthine in FF in patients with EM, which is similar to previous studies,^27^ suggesting that purine metabolism was disturbed in patients with EM and participated in the occurrence and development of this disease. Some studies have found that purine metabolites regulate oocyte meiosis, suggesting that the decline in oocyte quality in the EM group may be related to the upregulation of purine metabolism.^28^

Researchers have observed that the concentrations of phospholipids and lactic acid in the EM group increased significantly, while the concentrations of fatty acids, choline, glucose, aspartic acid alanine, lysine, leucine, valine, proline, and phosphocholine decreased significantly.^22^ A recent metabolomics study by NMR found increased levels of lactic acid, glucose, pyruvate and valine in patients with endometriosis.^29^ In this study, we observed the upregulation of L‐histidine, L‐proline, L‐tryptophan, L‐phenylalanine, L‐tyrosine, glutamyl arginine, glutamine, glutamyl phenylalanine, and their derivatives. These metabolites were mainly related to the metabolism of aromatic amino acids and glutathione. It was also found that the contents of indole metabolites such as indole, dihydroindole, indole lactic acid, indole‐3‐acetamide, and 5‐hydroxyindole acetic acid increased, which may come from tryptophan metabolism. Tryptophan derivatives such as indole can signal exogenous receptors, including aryl hydrocarbon receptors, to induce tolerance. The upregulation of tryptophan and related indole derivatives in EM may be one of the reasons for ectopic endometrial escape from autoimmune attack.^30^ Some researchers found that compared with the infertile control group, the concentrations of serum glutathione and superoxide dismutase (SOD) in the endometriosis group were higher, indicating an increase in systemic and FF oxidative stress in infertile patients with endometriosis.^31^ In this study, we also found the upregulation of glutathione pathway metabolites, indicating that there may be an upregulation of oxidative stress in FF in the EM group, which supports previous studies. However, some targeted metabolomics studies found no differential features in the FF of patients.^32^, ^33^ This may be due to the limited scan range relying on the metabolite standards of the targeted metabolomics.

It was also observed that some lipid‐related metabolites were upregulated in the EM group. 9,12,13‐Trihydroxy‐10‐octadecenoic acid is a trihydroxy octadecenoic acid metabolite of linoleic acid. Vascular tissues convert various polyunsaturated fatty acids into monohydroxy and trihydroxy metabolites derived from hydroperoxides, which may be involved in regulating the synthesis of prostaglandins.^34^ It was suggested that there may be an upregulation of the inflammatory response and oxidative stress in the FF of patients with EM. N‐oleoylethanolamine and oleamide are fatty amides that were upregulated in the EM group in this study. Fatty amides can be used as endogenous signaling molecules. In addition to their anti‐inflammatory activities, fatty amides may also interact with a variety of neurotransmitter systems to exert analgesic and neuroprotective effects.^35^, ^36^ For example, oleamides are structurally related to the endogenous cannabinoid anandamide and have the ability to bind to cannabinoid receptor‐1 (CB1) receptors as complete agonists. These fatty amides were upregulated in FF in the EM group, which is consistent with the common phenomenon of dysmenorrhea and upregulation of local inflammatory levels in patients with EM. It has been found that the cannabinoid receptor CB1 contributes to the development of ectopic lesions in mouse models of endometriosis. Therefore, how to regulate the level of cannabinoids in women with endometriosis may be a key research area for the potential development of new therapies for the disease.^37^, ^38^

Our findings showed that lipids such as LPC 18:0, LPC 15:0, LPC 18:3, LPC 20:1, LPC 20:0, LPC 14:0, LPC 17:0, lysophosphatidic acids (LPA) 20:4, PC 36:4, PC 38:4, PC 36:6, and phosphatidyl inositol (PI) 38:4 were downregulated in the EM group, which is consistent with previous studies.^16^, ^29^, ^39^, ^40^ However, other studies compared the FF lipid metabolism of 10 patients with endometriosis with 10 controls and found that the concentrations of sphingolipids and phosphatidylcholine in the EM group increased.^22^ The difference in these results may be due to the small sample size. Due to lipid peroxidation in patients with endometriosis, the total lipid levels in FF decrease. The oxidative intensity of free radicals is significantly increased, and the antioxidant protection activity is simultaneously decreased, leading to the accumulation of toxic products of lipid peroxidation. This compromises the integrity of the cell membrane, ion permeability, and energy mechanism and leads to cell dysfunction.^41^ It is well known that these mechanisms lead to the spread and progression of endometriosis.^41^ PC is one of the main sources of polyunsaturated fatty acids. It has a variety of biological activities and can produce LPC under the catalysis of enzymes.^42^ There is evidence that PC contributes to proliferative growth and programmed cell death.^43^ PC synthesis is increased by fatty acids and fatty acid‐derived substrates, which are often observed in cancer cells. In contrast to malignant tumors, PC in the endometrium of endometriosis decreased significantly. This difference can be used to distinguish between endometriosis and malignant tumors.^43^ PCs are the source of sphingomyelin and prostaglandins, which mediate inflammation in the pathophysiology of endometriosis. In addition, PC is closely related to the inflammatory process.^44^ Our study also found that LPC was negatively correlated with the oocyte maturation rate and high‐quality embryo rate. Therefore, PC and LPC have been identified as potential biomarkers of endometriosis.

In this study, it was also observed that several medium‐ and short‐chain acyl‐carnitines were downregulated in the EM group. Acyl‐carnitine, which is composed of fatty acids esterified into carnitine molecules, is an intermediate oxidative metabolite with proinflammatory properties. The function of carnitine is to oxidize long‐chain fatty acids through the mitochondrial membrane. In some cases, when fatty acids are not fully metabolized, long‐chain fatty acids CoA accumulate. It promotes the conversion to acyl‐carnitine, which can be exported from mitochondria and cells.^45^ Therefore, the abnormal level of acyl‐carnitine is a reflection of imbalance. Octenoyl‐L‐carnitine, as medium‐chain acyl‐carnitine, is formed by esterification with L‐carnitine or by peroxisome metabolism of longer‐chain acyl‐carnitine.^46^ Propionyl‐carnitine is classified as short‐chain acyl‐carnitine. It is one of the most abundant carnitine groups in the body.^47^ In previous studies, decreased acyl‐carnitine levels were found in peritoneal fluid in patients with endometriosis,^39^ and plasma levels of long‐chain acyl‐carnitine were higher and short‐chain carnitine levels were lower in EM patients.^12^ The downregulation of a variety of short‐chain acyl‐carnitines in FF in the EM group suggested that changes in acyl‐carnitine metabolism may be involved in the occurrence and development of EM. This also proves that the effect of oxidative stress and inflammatory reactions on the oocyte quality of endometriosis patients deserves further discussion.

In addition, differences in some other metabolites were observed. Beta‐D‐glucose 6‐phosphate and glucose 1‐phosphate were found to be upregulated in the FF of the EM group in this study, suggesting that there were changes in glucose metabolism in patients with endometriosis. This will help us to gain a deeper understanding of the changes in the glucose metabolism process of endometriosis. Progesterone is necessary for embryo implantation, pregnancy maintenance and breast tissue development for milk production. During implantation and pregnancy, progesterone seems to reduce the maternal immune response to allow pregnancy. A decrease in progesterone levels may be a step in promoting delivery.^48^ The downregulation of progesterone in the EM group may be one of the reasons for the low implantation rate of EM patients. However, this study did not explore the embryo implantation rate, so we did not find a relationship between this index and clinical indicators. Bilirubin is a yellow bile pigment, a degradation product of heme, and a kind of antioxidant. In this study, bilirubin was downregulated in FF in the EM group, and there was a significant positive correlation between bilirubin and the high‐quality embryo rate. This is contrary to the increase in bilirubin in cyst fluid of the EM group found by previous researchers.^49^ However, some studies have found that there is no difference in blood bilirubin in patients with endometriosis,^50^ which may be due to the difference in the samples. As an antioxidant, the downregulation of bilirubin may indicate an increase in oxidative stress in FF, indicating that EM may enhance the level of oxidative stress. Ketoleucine is an abnormal metabolite caused by incomplete breakdown of branched‐chain amino acids and is a metabolic toxin. Previous studies have focused on maple syrup urine disease (MSUD).^51^ In this study, we found for the first time that ketoleucine levels were downregulated in the FF of EM patients, which may suggest a new related pathway.

This study provides new perspectives on metabolic changes in endometriosis, but there are still some limitations. One of the limitations is that ovarian stimulation with exogenous gonadotropin may alter follicular metabolomics. Due to the current personalized adjustment of our ovulation promotion program based on the specific situation of each patient, it is not possible to ensure that patients use a consistent level of exogenous gonadotropin stimulation. Therefore, we exclude the approximate impact by maintaining a consistent overall level between the two groups. Second, because not all of the patients included in this study received single embryo transfer, it is difficult to study the relationship between endometriosis and embryo implantation or pregnancy outcome. Future studies should focus on the impact of current results on clinical outcomes such as embryo implantation, clinical pregnancy, and live birth while continuing to attempt to compare and contrast the various phenotypes of endometriosis (endometrioma, peritoneal endometriosis, deeply infiltrating endometriosis). Third, although nontargeted metabolomics based on LC‒MS has the advantages of comprehensiveness and unbiasedness, there are still many metabolites that are difficult to accurately annotate, which will result in the loss of information and may introduce interference from exogenous substances. Finally, some differential metabolites and pathways found in this study deserve further study in the oocyte model to determine the specific mechanisms that affect oocyte quality.

## 4 CONCLUSIONS

In summary, this study characterized the differences in metabolites and related pathway profiles between infertile patients with endometriosis and controls. This article provides direct evidence for the mechanism of endometriosis leading to the decline of oocyte quality, which has been unclear clinically. The results obtained in this work can provide a better comprehensive understanding of the pathogenesis and progression of the disease and provide a new direction for the study of oocyte quality, as well as the use of specific biochemical markers for reliable laboratory diagnosis.

## 5 MATERIALS AND METHODS

### 5.1 Subjects

This is a prospective clinical study. A total of 50 participants who received in vitro fertilization and embryo transfer (IVF‐ET) or intracytoplasmic sperm injection and embryo transfer (ICSI‐ET) at the Reproductive Medicine Center, Renmin Hospital of Wuhan University, from December 2020 to January 2022, were recruited.

The inclusion criteria were as follows: (1) infertile women aged 22−40 years old; (2) normal ovarian function; (3) no operation within 3 months before FF collection; (4) body mass index (BMI) 18−25 kg/m^2^; and (5) the first cycle of IVF‐ET or ICSI‐ET.

Exclusion criteria: combined uterine adenomyosis; premature ovarian failure and other factors that may affect follicular development; abnormal uterine structure; polycystic ovary syndrome, diabetes, thyroid dysfunction, and other endocrine diseases; hepatitis B virus, human immunodeficiency virus (HIV), and other infectious diseases; cardiovascular diseases; dyslipidemia; systemic lupus erythematosus and other rheumatic diseases and autoimmune diseases.

The EM group was divided into two subgroups: the EMD group and the SEM group. The patients in the EMD group were diagnosed with EM by laparoscopy or laparotomy and were divided into stage III or IV according to the 1997 American Society for Reproductive Medicine staging. The SEM group consisted of patients diagnosed with EM by type B ultrasound. SEM patients choose to undergo assisted reproduction before laparoscopic surgery for fertility or other reasons.

The patients in the control group were infertile women due to tubal factors or male factors matched with the patients in the EM group by age, BMI, fertilization and ovulation and excluded EM (through medical history, symptoms, signs). Tubal factors included salpingectomy or salpingotomy performed after tubal ectopic pregnancy, tubal obstruction and tubal adhesion around or distorted caused by infection, tubal ligation, etc. All participants provided written informed consent. The Ethics Committee of Renmin Hospital of Wuhan University reviewed this study and approved the study (WDRY2018‐K009).

### 5.2 Follicular fluid collection

All participants adopted appropriate ovulation induction treatment to promote ovulation according to their personal conditions. In our research center, the first‐line treatment for endometriosis is the long GnRH‐α protocol. Follicular development and serum sex hormone levels were monitored regularly until the day of chorionic gonadotrophin (HCG, Lizhu, China) injection. In the process of ovulation induction, the drug dosage was adjusted according to follicular development and hormone levels.

During regular monitoring of ovulation, when at least two follicles in the bilateral ovaries were larger than 18 mm, 10,000 U HCG was injected intramuscularly to induce the final maturation of the follicles. Thirty‐four to thirty‐five hours after HCG injection, oocytes and FF were collected by puncturing mature follicles larger than 18 mm under ultrasound guidance. The oocytes were cultured in the medium, and the FF was transferred to the Eppendorf tube (EP tube). The collected FF was centrifuged at 4°C and 15,000 rpm for 10 min. The supernatant was cryopreserved at −80°C for further analysis.

### 5.3 Fertilization and embryo quality assessment

Oocytes were fertilized and transferred to a new medium 3 h after collection. The occurrence of 2PN was observed and recorded. The evaluation criteria for the quality of cleavage embryos were according to the Vienna Consensus.^52^ High‐quality embryos at the cleavage stage were defined as follows: D3 embryos were grade I and II, and the number of blastomeres was 6−9.

### 5.4 Sample preparation

Each FF sample was thawed on ice and vortexed for 30 s. A 100 µL sample was transferred to an EP tube, 400 µL methanol (Fisher Chemical, Canada, cat. no. A452‐4) was added, and the tube was vortexed for 1 min. Then, the mixture was centrifuged at 15,000 rpm for 10 min at 4°C, and the supernatant (400 µL) was collected. After freezing overnight at −20°C, the samples were centrifuged at 15,000 rpm for 10 min at 4°C, and the supernatant (300 µL) was dried with N_2_ for preservation. Before injection, the dried samples were mixed with 80 µL 80% methanol and centrifuged at 15,000 rpm for 10 min at 4°C. The supernatant (60 µL) was transferred to a fresh glass vial for LC‒MS analysis. QC samples were prepared by mixing 5 µL of the supernatant from each sample to assess the stability of the analytical method.

### 5.5 LC‒MS analysis and data acquisition

LC‒MS analysis was performed using an Ultimate 3000 liquid system (Thermo Scientific, America) coupled with a timsTOF Pro mass spectrometer (Bruker Daltonics, Germany). Two chromatographic separation methods (RPLC and HILIC) were used for nontargeted metabolomics analysis to improve the quality and quantity of metabolite identification for hydrophilic and hydrophobic metabolites.

MS data were collected using a timsTOF Pro mass spectrometer with a top 3 data‐dependent acquisition (DDA) method and trapped ion mobility spectrometry (TIMS) off in positive and negative modes. Specific chromatographic separation method parameters and MS parameters were shown in our previously published article.^53^

### 5.6 Statistical analysis

Raw data from LC‒MS were processed using MetaboScape software 4.0 (Bruker Daltonics). The T‐ReX at the 3D feature finding algorithm was applied for feature detection, deconvolution, alignment, and integration. The intensity threshold of feature detection was set as 2000 counts, and the minimum peak length was 10 spectra. The in‐house database, Human Metabolome Database (HMDB, http://www.hmdb.ca/),^54^ NIST Mass Spectral Library (https://chemdata.nist.gov/), MassBank of North America (MoNA, https://mona.fiehnlab.ucdavis.edu/), and Bruker MetaboBASE Personal Library 3.0 (Bruker Daltonics) were used for metabolite identification.

The Shapiro‒Wilk test and Bartlet's test were applied to test the normality and homogeneity of variances of each feature. The *p* value was computed by Student's *t* test or the Mann‒Whitney test depending on the type of data and adjusted by the Benjamini‒Hochberg method. Then, Metaboanalyst 5.0 (https://www.metaboanalyst.ca/) was used for pathway enrichment analysis. The Pearson's correlation coefficient between differential metabolites and the Spearman's correlation coefficient between differential metabolites and the ovarian function index were calculated using the psych package in R. The correlation network was plotted by Cytoscape 3.9.1. The R package random forest was used to select important metabolites and build a random forest model to distinguish the EM group from the control group. PCA, PLS‐DA and OPLS‐DA were performed with SIMCA software after autoscaling.

## AUTHOR CONTRIBUTIONS

YQW, JNL, YZ, and TLY conceived the original ideas. YQW and JNL collected samples and clinical data. YQW, ZRZ, and XQR conducted the metabolomics analysis. ZRZ performed the statistical analysis. YQW interpreted the data. YQW, ZRZ, and YYZ cowrote the manuscript. QQW and YYZ provided critical feedback and helped shape the manuscript. YJZ, SMC, and YZ supervised and revised the manuscript. All authors approved the final version of the manuscript for publication.

## CONFLICT OF INTEREST STATEMENT

The authors report no declarations of interest.

## ETHICS STATEMENT

Written informed consent was obtained from all participants. The Ethics Committee of Renmin Hospital of Wuhan University reviewed this study and approved the study (WDRY2018‐K009).

## Supporting information

Supporting Information

## Data Availability

The metabolomics data generated in this study were deposited in the ProteomeXchange Consortium (https://www.iprox.org/). Project number: PXD034813. Source data were provided with this article.
