# Supplementary material for: Nontargeted metabolomics analysis of follicular fluid in patients with endometriosis provides a new direction for the study of oocyte quality
Source: MedComm (2020). 2023 May 30;4(3):e302. doi: 10.1002/mco2.302 (PMC10229744; doi:10.1002/mco2.302)
Supplement: Supplementary file 1 — Supporting Information [file MCO2-4-e302-s001.docx]

**Nontargeted metabolomics analysis of follicular fluid in patients with endometriosis** **provides a new direction for the study of oocyte quality**

Yiqiu Wei ^1#^, Zhourui Zhang^2#^, Yaoyao Zhang^3#^, Jianan Li^1^, Xianqin Ruan^2^, Qiongqiong Wan^2^, Tailang Yin^1^, Yujie Zou^1*^, Suming Chen^2*^, Yan Zhang^4*^

^1^ Reproductive Medicine Center, Renmin Hospital of Wuhan University, Wuhan, Hubei 430060, China.

^2^ The Institute for Advanced Studies, Wuhan University, Wuhan, Hubei 430072, China.

^3^ Department of Obstetrics and Gynecology, Key Laboratory of Birth Defects and Related of Women and Children of Ministry of Education, West China Second University Hospital, Sichuan University, Chengdu, Sichuan 610041, China

^4^ Department of Clinical Laboratory, Renmin Hospital of Wuhan University, Wuhan, Hubei 430060, China.

*** Correspondence:**

Yan Zhang, [peneyyan@mail.ustc.edu.cn](mailto:peneyyan@mail.ustc.edu.cn)

Suming Chen, [sm.chen@whu.edu.cn](mailto:sm.chen@whu.edu.cn)

Yujie Zou, [yujie.zou@whu.edu.cn](mailto:yujie.zou@whu.edu.cn)

**Supplementary Figures**


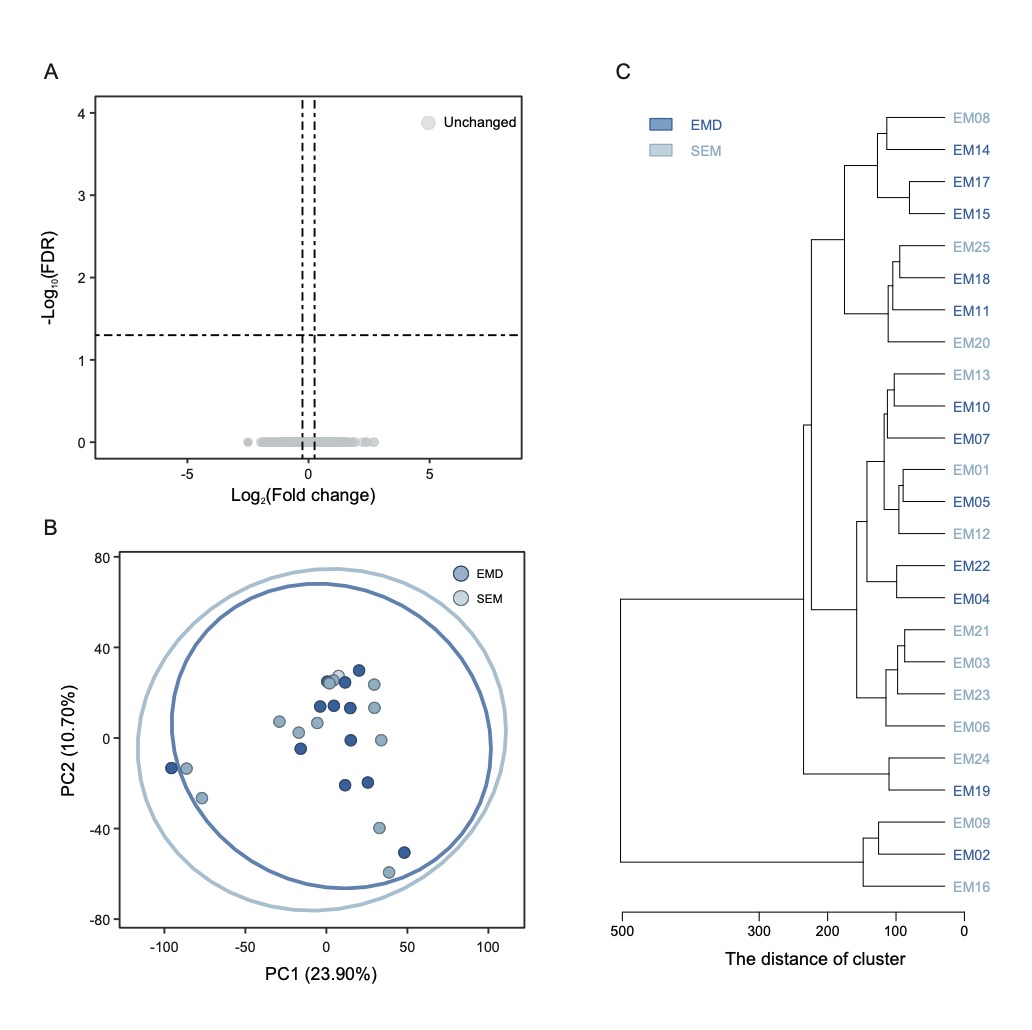


Figure S1: Subgroup analysis. (A) Volcano plot analysis between the EMD and SEM subgroups, with no differential metabolites. (B) PCA between the two subgroups. (C) Cluster analysis between the two subgroups.


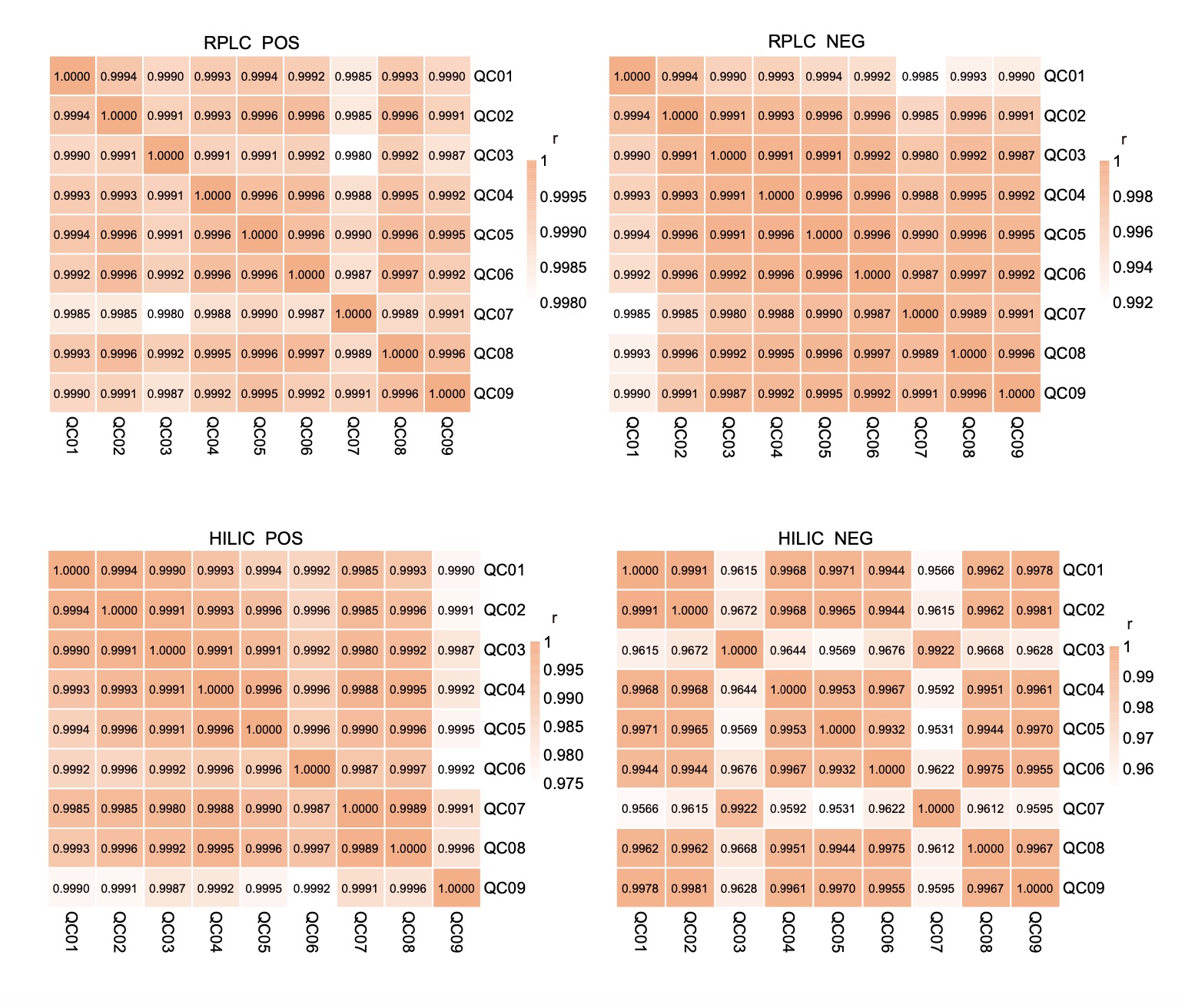


Figure S2: Stability analysis. The correlation analysis matrix of all QCs in the four modes.


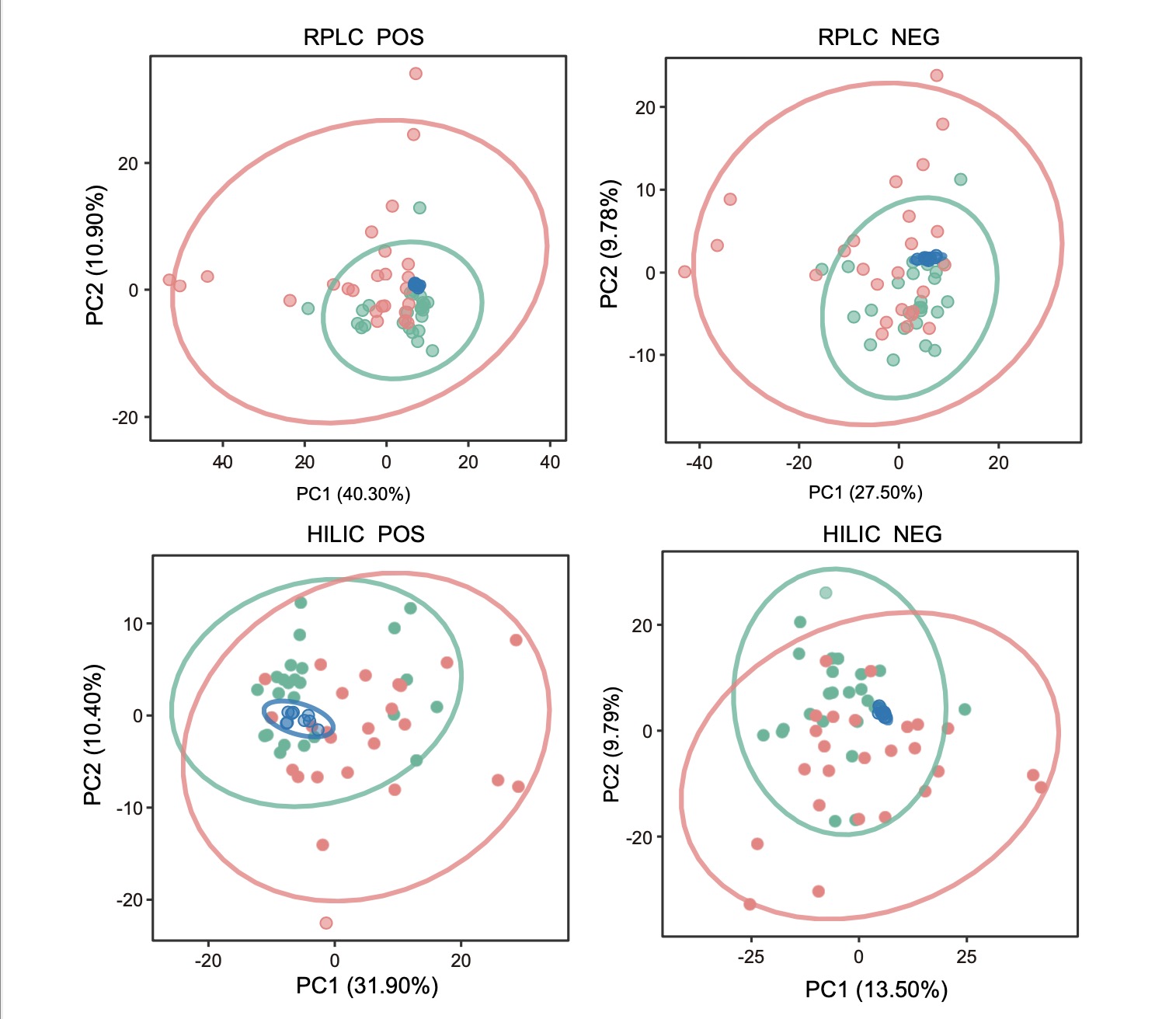


Figure S3: PCA in four modes.


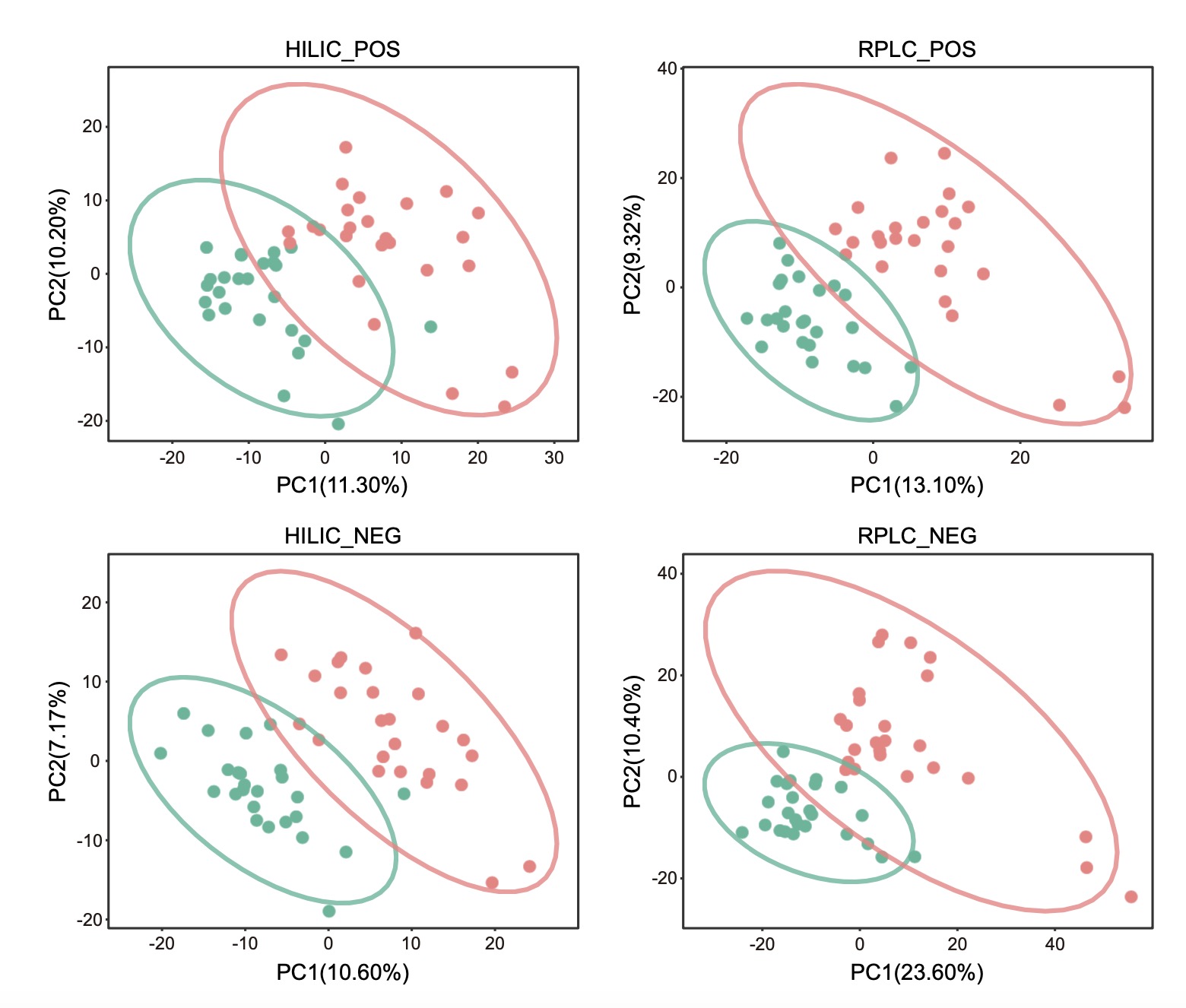


Figure S4: PLS-DA in four modes.


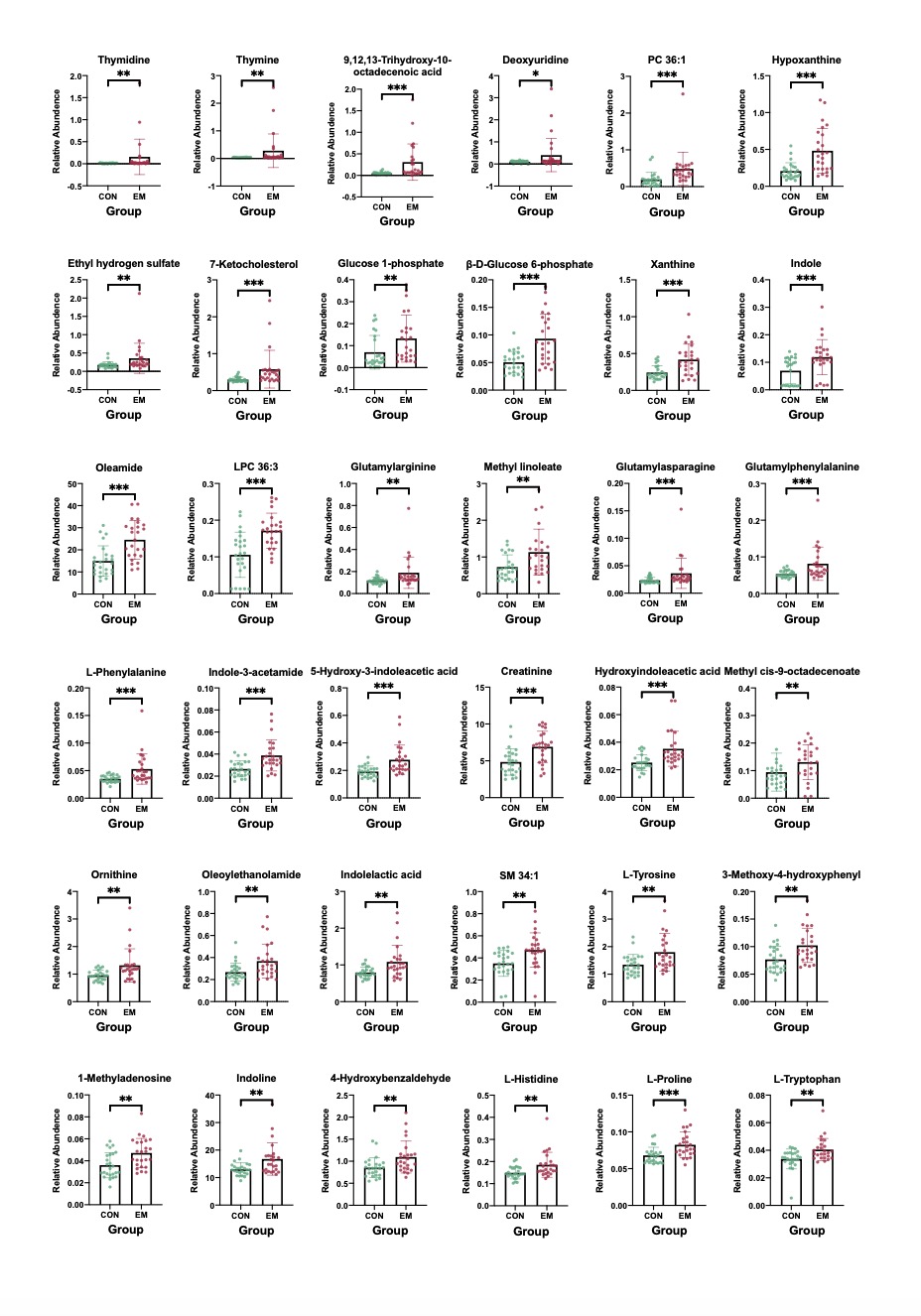


Figure S5: Histogram of upregulated metabolites. The EM group is displayed in red, and the control group is displayed in green. The y-axis represents the integrated data of mass spectral intensity normalized by TIC. * *P* <0.05, ** *P* <0.01, *** *P* <0.001.


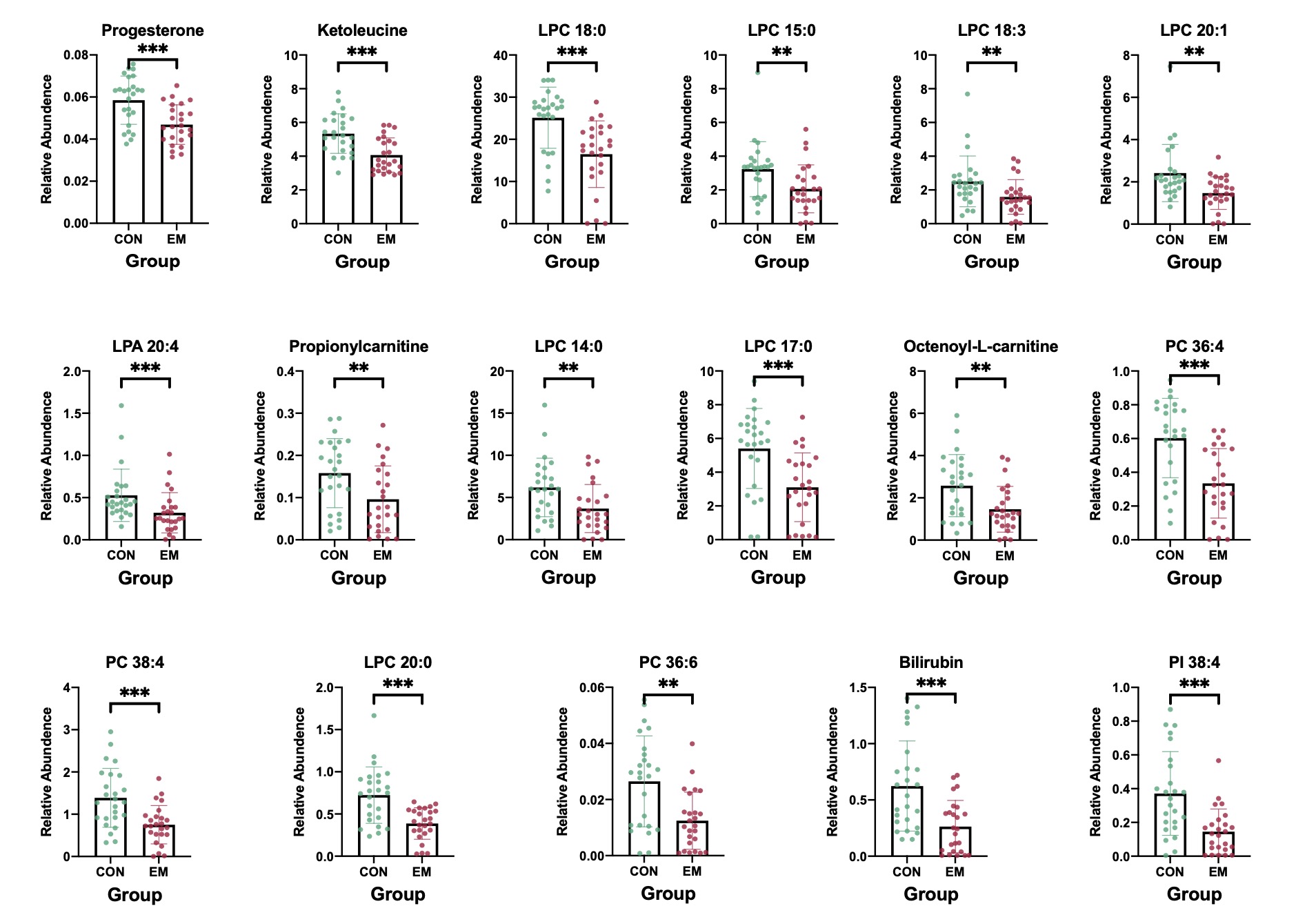


Figure S6: Histogram of downregulated metabolites. The EM group is displayed in red, and the control group is displayed in green. The y-axis represents the integrated data of mass spectral intensity normalized by TIC. * *P* <0.05, ** *P* <0.01, *** *P* <0.001.


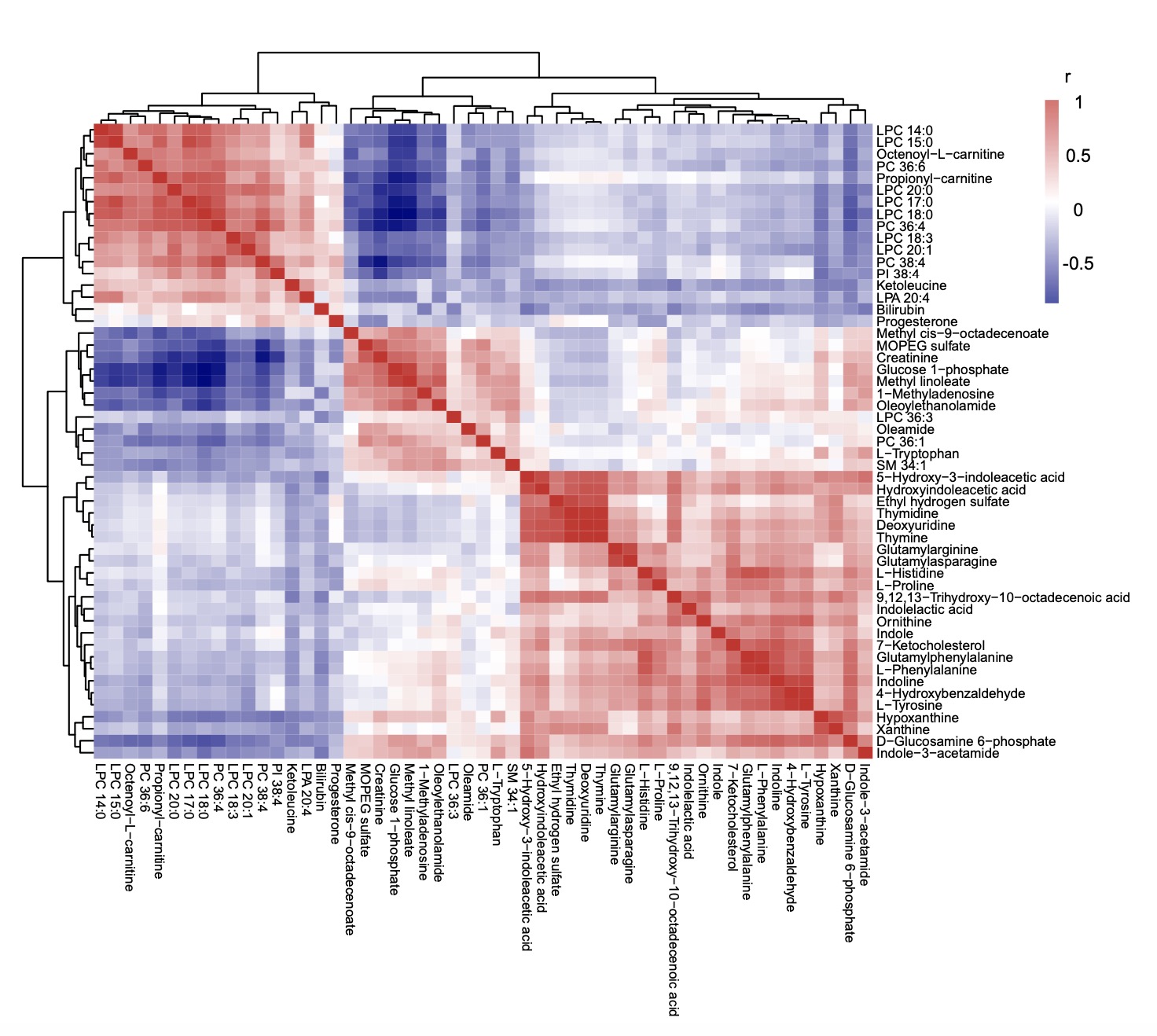


Figure S7: Overall correlation analysis of differential metabolites.

**Supplementary Tables**

**Table S1** Differential metabolites in FF detected by LC‒MS/MS

| **ID** | **Metabolities** | **HMDB ID** | **RT [min]** | **m/z** | **Adduct Type** | **Formula** | **Relative abundance** | | **Log2FC(EM/CON)** | ***P* value** | **FDR** | **Log10(FDR)** |
| --- | --- | --- | --- | --- | --- | --- | --- | --- | --- | --- | --- | --- |
|  |  |  |  |  |  |  | **CON** | **EM** |  |  |  |  |
| HIN0266 | MOPEG sulfate | HMDB0000559 | 1.10 | 263.02292 | [M-H]- | C9H12O7S | 0.0767 | 0.1023 | 0.42 | 2.13E-03 | 0.0372 | 1.43 |
| RPN0467 | 9,12,13-Trihydroxy-10-octadecenoic acid | HMDB0038555 | 9.97 | 329.23295 | [M-H]- | C18H34O5 | 0.0503 | 0.3085 | 2.62 | 2.40E-04 | 0.0143 | 1.84 |
| RPP0160 | 1-Methyladenosine | HMDB0003331 | 1.18 | 282.11979 | [M+H]+ | C11H15N5O4 | 0.0360 | 0.0471 | 0.39 | 2.77E-03 | 0.0214 | 1.67 |
| RPP0104 | 4-Hydroxybenzaldehyde | HMDB0011718 | 0.82 | 123.04411 | [M+H]+ | C7H6O2 | 0.8562 | 1.0956 | 0.36 | 5.15E-03 | 0.0320 | 1.49 |
| HIN0205 | Ketoleucine | HMDB0000695 | 0.89 | 129.05551 | [M-H]- | C6H10O3 | 5.3403 | 4.0713 | -0.39 | 2.40E-04 | 0.0097 | 2.01 |
| RPP0163 | 5-Hydroxy-3-indoleacetic acid | HMDB0000763 | 1.33 | 192.06561 | [M+H]+ | C10H9NO3 | 0.1917 | 0.2778 | 0.54 | 1.55E-04 | 0.0030 | 2.52 |
| HIP0051 | 7-Ketocholesterol | HMDB0000501 | 0.57 | 401.34166 | [M+H]+ | C27H44O2 | 0.2998 | 0.5794 | 0.95 | 2.77E-05 | 0.0060 | 2.22 |
| RPP1450 | Bilirubin | HMDB0000054 | 17.59 | 585.2698 | [M+H]+ | C33H36N4O6 | 0.6232 | 0.2631 | -1.24 | 4.71E-04 | 0.0062 | 2.21 |
| HIP0304 | Creatinine | HMDB0000562 | 2.94 | 114.06609 | [M+H]+ | C4H7N3O | 4.8425 | 6.9006 | 0.51 | 5.26E-04 | 0.0185 | 1.73 |
| HIN0341 | Deoxyuridine | HMDB0000012 | 1.74 | 227.0672 | [M-H]- | C9H12N2O5 | 0.1167 | 0.4620 | 1.98 | 1.61E-03 | 0.0322 | 1.49 |
| HIN1148 | D-Glucosamine 6-phosphate | HMDB0001254 | 9.29 | 258.03848 | [M-H]- | C6H14NO8P | 0.0505 | 0.0932 | 0.89 | 1.69E-04 | 0.0083 | 2.08 |
| HIP1174 | Glucose 1-phosphate | HMDB0001586 | 9.67 | 261.03669 | [M+H]+ | C6H13O9P | 0.0700 | 0.1326 | 0.92 | 2.65E-03 | 0.0410 | 1.39 |
| HIN0502 | Ethyl hydrogen sulfate | HMDB0031233 | 3.90 | 124.9912 | [M-H]- | C2H6O4S | 0.1762 | 0.3567 | 1.02 | 2.47E-03 | 0.0404 | 1.39 |
| HIP1248 | Glutamylarginine | HMDB0028813 | 10.36 | 304.16114 | [M+H]+ | C11H21N5O5 | 0.1194 | 0.1893 | 0.67 | 2.00E-03 | 0.0361 | 1.44 |
| HIP1192 | Glutamylasparagine | HMDB0028814 | 9.78 | 262.1031 | [M+H]+ | C9H15N3O6 | 0.0235 | 0.0362 | 0.62 | 6.49E-04 | 0.0211 | 1.68 |
| RPP0230 | Glutamylphenylalanine | HMDB0029156 | 5.42 | 295.12905 | [M+H]+ | C14H18N2O5 | 0.0537 | 0.0815 | 0.60 | 7.59E-04 | 0.0086 | 2.06 |
| RPN0104 | Hydroxyindoleacetic acid | HMDB0000763 | 1.32 | 190.0505 | [M-H]- | C10H9NO3 | 0.0252 | 0.0353 | 0.48 | 1.69E-04 | 0.0130 | 1.89 |
| HIP0325 | Hypoxanthine | HMDB0000157 | 3.07 | 137.04574 | [M+H]+ | C5H4N4O | 0.2068 | 0.4819 | 1.22 | 3.39E-05 | 0.0063 | 2.20 |
| RPP0166 | Indole | HMDB0000738 | 1.39 | 118.06514 | [M+H]+ | C8H7N | 0.0693 | 0.1187 | 0.78 | 9.57E-04 | 0.0100 | 2.00 |
| RPP0142 | Indole-3-acetamide | HMDB0029739 | 1.02 | 175.08678 | [M+H]+ | C10H10N2O | 0.0266 | 0.0388 | 0.55 | 2.62E-04 | 0.0043 | 2.37 |
| RPN0229 | Indolelactic acid | HMDB0000671 | 7.01 | 204.06623 | [M-H]- | C11H11NO3 | 0.7948 | 1.0850 | 0.45 | 2.30E-03 | 0.0482 | 1.32 |
| RPP0177 | Indoline | HMDB0253472 | 1.44 | 120.08082 | [M+H]+ | C8H9N | 13.0320 | 16.7526 | 0.36 | 5.50E-03 | 0.0332 | 1.48 |
| HIP1235 | L-Histidine | HMDB0000177 | 10.10 | 156.07674 | [M+H]+ | C6H9N3O2 | 0.1490 | 0.1854 | 0.32 | 3.04E-03 | 0.0454 | 1.34 |
| RPN1091 | LPA 20:4 | NA | 15.34 | 457.23556 | [M-H]- | C23H39O7P | 0.5282 | 0.3210 | -0.72 | 7.59E-04 | 0.0255 | 1.59 |
| RPP0752 | LPC 14:0 | NA | 12.82 | 468.30846 | [M+H]+ | C22H46NO7P | 6.1872 | 3.6961 | -0.74 | 9.01E-03 | 0.0469 | 1.33 |
| RPP0843 | LPC 15:0 | NA | 13.48 | 482.32416 | [M+H]+ | C23H48NO7P | 3.2335 | 2.0650 | -0.65 | 4.24E-03 | 0.0277 | 1.56 |
| RPP1053 | LPC 17:0 | NA | 14.79 | 510.35549 | [M+H]+ | C25H52NO7P | 5.4061 | 3.1068 | -0.80 | 5.10E-04 | 0.0065 | 2.19 |
| RPP1109 | LPC 18:0 | NA | 15.18 | 524.37104 | [M+H]+ | C26H54NO7P | 25.1277 | 16.4738 | -0.61 | 1.08E-04 | 0.0024 | 2.62 |
| RPP0760 | LPC 18:3 | NA | 12.94 | 518.32398 | [M+H]+ | C26H48NO7P | 2.5080 | 1.5874 | -0.66 | 5.86E-03 | 0.0347 | 1.46 |
| RPP1343 | LPC 20:0 | NA | 16.68 | 552.40232 | [M+H]+ | C28H58NO7P | 0.7236 | 0.3872 | -0.90 | 1.85E-04 | 0.0036 | 2.45 |
| RPP1185 | LPC 20:1 | NA | 15.65 | 550.38663 | [M+H]+ | C28H56NO7P | 2.4171 | 1.4692 | -0.72 | 3.25E-03 | 0.0236 | 1.63 |
| RPP1407 | LPC 36:3 | NA | 17.26 | 770.60481 | [M+H]+ | C44H84NO7P | 0.1056 | 0.1713 | 0.70 | 1.10E-04 | 0.0024 | 2.61 |
| RPP0231 | L-Phenylalanine | HMDB0000159 | 5.42 | 166.0864 | [M+H]+ | C9H11NO2 | 0.0352 | 0.0529 | 0.59 | 5.53E-04 | 0.0068 | 2.17 |
| HIP1144 | L-Proline | HMDB0000162 | 9.14 | 116.07057 | [M+H]+ | C5H9NO2 | 0.0681 | 0.0824 | 0.28 | 5.99E-04 | 0.0205 | 1.69 |
| RPP0194 | L-Tryptophan | HMDB0000929 | 3.03 | 227.07865 | [M+Na]+ | C11H12N2O2 | 0.0337 | 0.0405 | 0.26 | 4.24E-03 | 0.0277 | 1.56 |
| RPP0099 | L-Tyrosine | HMDB0000158 | 0.82 | 182.08123 | [M+H]+ | C9H11NO3 | 1.3490 | 1.8022 | 0.42 | 6.24E-03 | 0.0362 | 1.44 |
| RPP1483 | Methyl linoleate | HMDB0034381 | 17.97 | 295.2634 | [M+H]+ | C19H34O2 | 0.7297 | 1.1334 | 0.64 | 7.51E-03 | 0.0413 | 1.38 |
| RPP1277 | Methyl cis-9-octadecenoate | HMDB0254581 | 16.30 | 297.27905 | [M+H]+ | C19H36O2 | 0.0946 | 0.1308 | 0.47 | 7.51E-03 | 0.0413 | 1.38 |
| RPP0330 | Octenoyl-L-carnitine | HMDB0240723 | 7.98 | 286.20146 | [M+H]+ | C15H27NO4 | 2.5796 | 1.4621 | -0.82 | 6.24E-03 | 0.0362 | 1.44 |
| RPP1284 | Oleamide | HMDB0002117 | 16.35 | 282.27939 | [M+H]+ | C18H35NO | 14.9588 | 24.5657 | 0.72 | 6.74E-05 | 0.0016 | 2.78 |
| RPP1231 | Oleoylethanolamide | HMDB0002088 | 15.95 | 326.3056 | [M+H]+ | C20H39NO2 | 0.2680 | 0.3683 | 0.46 | 6.24E-03 | 0.0362 | 1.44 |
| HIP1240 | Ornithine | HMDB0000214 | 10.15 | 133.09719 | [M+H]+ | C5H12N2O2 | 0.9573 | 1.3174 | 0.46 | 2.15E-03 | 0.0378 | 1.42 |
| RPP1656 | PC 36:1 | NA | 21.79 | 788.61435 | [M+H]+ | C44H86NO8P | 0.1938 | 0.4826 | 1.32 | 8.57E-06 | 0.0004 | 3.45 |
| HIN0428 | PC 36:4 | NA | 2.91 | 840.57404 | [M+CH3COO]- | C44H80NO8P | 0.6028 | 0.3344 | -0.85 | 8.42E-05 | 0.0059 | 2.23 |
| RPP1569 | PC 36:6 | NA | 19.05 | 778.53842 | [M+H]+ | C44H76NO8P | 0.0265 | 0.0125 | -1.09 | 2.84E-03 | 0.0215 | 1.67 |
| HIP0291 | PC 38:4 | NA | 2.82 | 810.59923 | [M+H]+ | C46H84NO8P | 1.3876 | 0.7509 | -0.89 | 4.34E-04 | 0.0166 | 1.78 |
| HIN0600 | PI 38:4 | NA | 4.71 | 885.54788 | [M-H]- | C47H83O13P | 0.3713 | 0.1454 | -1.35 | 2.62E-04 | 0.0097 | 2.01 |
| HIP1197 | Progesterone | HMDB0001830 | 9.79 | 315.23142 | [M+H]+ | C21H30O2 | 0.0585 | 0.0469 | -0.32 | 2.87E-04 | 0.0138 | 1.86 |
| RPP0139 | Propionyl-carnitine | HMDB0000824 | 0.98 | 218.13885 | [M+H]+ | C10H19NO4 | 0.1580 | 0.0960 | -0.72 | 8.95E-03 | 0.0469 | 1.33 |
| RPP1304 | SM 34:1 | NA | 16.49 | 703.57494 | [M+H]+ | C39H79N2O6P | 0.3505 | 0.4722 | 0.43 | 1.11E-03 | 0.0111 | 1.95 |
| HIP0232 | Thymidine | HMDB0000273 | 1.50 | 243.09741 | [M+H]+ | C10H14N2O5 | 0.0113 | 0.1573 | 3.80 | 1.20E-03 | 0.0284 | 1.55 |
| HIP0231 | Thymine | HMDB0000262 | 1.50 | 127.05021 | [M+H]+ | C5H6N2O2 | 0.0324 | 0.2806 | 3.12 | 1.39E-03 | 0.0307 | 1.51 |
| HIN0499 | Xanthine | HMDB0000292 | 3.88 | 151.02589 | [M-H]- | C5H4N4O2 | 0.2429 | 0.4177 | 0.782384196 | 9.57E-04 | 0.0215 | 1.67 |

**Table S2. The pathways mainly involved in differential metabolites**

| **Pathway** | **hits** | **P** | **-Log10P** |
| --- | --- | --- | --- |
| Phenylalanine, tyrosine and tryptophan biosynthesis | 2 | 0.0028 | 2.5482 |
| Aminoacyl-tRNA biosynthesis | 5 | 0.0035 | 2.4522 |
| Phenylalanine metabolism | 2 | 0.0195 | 1.7100 |
| Pyrimidine metabolism | 3 | 0.0538 | 1.2692 |

**Table S3 Machine learning model evaluation**

|  | All | TOP20 | TOP10 |
| --- | --- | --- | --- |
| Accuracy | 0.878 | 0.946 | 0.904 |
| Sensitivity | 0.884 | 0.948 | 0.904 |
| Specificity | 0.872 | 0.944 | 0.904 |
| Precision | 0.890309524 | 0.955452381 | 0.919809524 |
| Recall | 0.884 | 0.948 | 0.904 |
| AUC | 0.9536 | 0.9888 | 0.9752 |
